# The epistemic challenges of CTOs: Commentary on . . . Community treatment orders†

**DOI:** 10.1192/pb.bp.113.045732

**Published:** 2014-02

**Authors:** Edwina Light

**Affiliations:** 1 University of Sydney, Australia

## Abstract

Controversy around the use of community treatment orders (CTOs) arises in part from their ambiguous evidence base. Recent research has provided valuable new insights into the effects of CTOs, while also highlighting the critical importance of first understanding what CTOs are and what they are meant to achieve. A genuine public discourse on the significance of CTOs will have multiple perspectives. This necessitates a more pluralistic approach to constructing the necessary knowledge of CTOs to enable communities to make sound decisions about their use.

Despite the controversies surrounding their efficacy, the utilisation of community treatment orders (CTOs) is increasing worldwide.^1,2^ Debates about ethical and human rights issues relating to CTOs span all jurisdictions, whether CTOs are a recent development (as in the 2007 Mental Health Act provisions in England and Wales) or more established (as in the provisions first introduced in the Australian state of New South Wales in 1990). These debates concern the principle of using coercion in clinical practice, the impact of CTOs on the autonomy and privacy interests of individuals, and the provision of appropriate mental healthcare that is not reliant on unnecessary compulsion to compensate for under-resourced community services.^3–6^ Such issues are common to all jurisdictions, despite the variations in legislative provisions for CTO systems, which include differences in the criteria for applying CTOs and the powers given to healthcare providers. These concerns are highlighted by the significant variation in the rates of use of CTOs around the world,^7^ which has prompted concerns about their use being determined by various factors besides clinical need.^8,9^

The controversy arises, in part, from the inconsistent results of research into CTOs, which has failed to demonstrate positive clinical outcomes and which reveals that patients, carers and clinicians have mixed views about CTOs.^4,10,11^ Although existing research into CTOs may be accurately representing variation and inconsistencies in their application, the heterogeneity and inadequacies of the evidence base^10,12^ make it difficult to draw conclusions from the published literature. Nevertheless, it is important to take proper account of the data that do exist.

## Effectiveness of CTOs in OCTET and other studies

The Oxford Community Treatment Order Evaluation Trial's (OCTET's) finding that CTOs did not reduce rates of hospital readmissions compared with existing provisions in England and Wales for supervised hospital leave^13^ is among a number of recent research outcomes providing valuable new insights into the effects of CTOs. OCTET was only the third randomised controlled trial in this area and the first with the opportunity to test existing supervision arrangements alongside the introduction of the new CTO system. Other recent research findings (from nonrandomised comparative,^14–16^ file audit and observational studies^17,18^) also suggest that CTOs may produce limited or specific outcomes. Community treatment orders are unlikely to reduce rates of hospital admission^15,16,18^ (an increasingly disputed measure of efficacy),^15,16,19^ but may reduce length of hospital stay among certain patient groups or where a CTO system is well established.^15,16,18^ They may reduce mortality associated with preventable physical illness^14^ and increase frequency of clinical contact.^15^ Recent surveys and qualitative studies continue to highlight mixed views among patients and clinicians about CTO uses and related clinical, ethical and human rights quandaries.^20,21^

## Asserting the purpose of CTOs and measuring their effects

Although more research on CTO outcomes and experiences is needed, it is also critically important that we understand what CTOs are and what they are meant to achieve. What are we trying to do by using CTOs? Avoid hospital readmissions? Reduce hospital stays? Stabilise people's health to enable illness insight or treatment engagement? Prioritise community-based over hospital-based coercive treatment? Prompt a health system response? Improve ‘quality of life’? As CTO systems evolve at policy and practice levels it could be argued that one or many of these are objectives sought when CTOs are used. For now, there is no clear consensus on their instrumental value.

In areas where it has been shown that CTOs do have some utility, it is often unclear whether these effects are due to the compulsory nature of CTOs or because of the intensity of the treatment they facilitate.^1,11,12^ Kisely *et al*,^14^ for example, note that the only consistent effect of CTOs is to increase the number of community and out-patient contacts, which is ‘a process rather than outcome measure’ and possibly the mechanism by which patient mortality was reduced in their study.^14^ Segal *et al*^16^ also highlighted the procedural nature of CTOs as a service delivery tool, not a treatment, and that the impact of a CTO is only as good as its execution, including the services provided.

For example, recent qualitative research examining the use of CTOs in New South Wales, Australia^22,23^ also bears out this idea – that CTOs are more of a reflection of mental healthcare services (in their entirety) than a simple therapeutic intervention. These studies found that the lived experience of CTOs for patients and carers was one of distress and profound ambivalence. This was associated with the experiences of severe mental illness as well as difficulties accessing the health system and with the implementation of CTOs. Many participants, along with many clinical and mental health review tribunal participants, described CTOs as being a safety net or trigger to access health services in a system that was often overstretched. In this way, a CTO was an order on both the patient and the health service, outlining mutual obligations for treatment adherence and appropriate treatment provision.

## What constitutes a fair assessment of healthcare?

What these, and other studies, remind us about research into mental health interventions and policies is that study design, the identification of outcome measures and the choice of measurement tools are all philosophical concerns. They reflect our ideas about what matters, about what (and who) counts, and about what counts as evidence. Thus, we should ask: should CTOs be determined as lacking in efficacy based on a disputed primary research finding of no difference in readmission rate in a small number of randomised controlled trials and other studies? To extend this logic is to argue that re-hospitalisation represents a treatment failure. Is it therefore reasonable to argue that in-patient care is always an undesirable outcome? This begs the question of the *telos* of mental healthcare. Does community mental health exist primarily to shield the hospital system from admissions?

The inherent frustration in conducting clinical research in mental healthcare is the lack of the kind of definitive outcomes found in internal medicine, such as survival or altered disease progression. If we desire a broader suite of benefits from interventions in the realm of mental healthcare, should we not use a variety of approaches to assess them? What are the benefits of any intervention in mental healthcare – reduced distress, improvement in function, life satisfaction, the capacity to partake of a life journey, reduced morbidity or less social disadvantage? How can these questions be best answered?

## CTO practice in the UK and Australia

In the meantime, the CTO system in England and Wales has provided an example of the benefits of a prominent and transparent public discourse about CTO policies and practices. Major academic projects, such as OCTET and its successors, and regulatory monitoring, such as that by the Care Quality Commission, provide publicly available data that allow for ongoing critical examination of involuntary treatment of people living with mental illness in the community. The Commission's *Monitoring the Mental Health Act in 2011/12* report^24^ shows that 4220 patients were subject to CTOs in 2011/2012, a 10% rise on the previous year, and that there is a wide variation in healthcare provider use of CTOs. This regular data collection and analysis, coupled with research projects, keeps the pressure on policy makers and practitioners to consider the reasons for rising numbers of CTOs and to develop appropriate policy and practice responses.

This provides a marked contrast to the situation in Australia where, despite long-established CTO schemes in each of its eight states and territories (some more than 20 years old), CTOs remain near invisible in mental health policy and public discourse. A recent review of national, state and territory mental health policies found almost no mention of CTOs, their organisation or role in the health system's care of people with mental illness.^6^ One state (Victoria) mental health strategy document was the only policy to detail a position on CTOs, stating that although ‘an important element of community-based treatment, their increasing use is of some concern’.^6^ Procedural information from state and territory tribunals and health departments provides data on activities under local mental health laws, such as numbers of CTOs made, however, these are not collected nationally or uniformly. A survey of state and territory tribunals and health departments for the most recent annual data found that rates in Australia are high (by world standards), increasing, and variable across states and territories (ranging from 30.2 per 100 000 in Tasmania to 98.8 per 100 000 in Victoria).^9^ This ‘invisibility’ of CTOs in Australian health policy has raised questions about the transparency and accountability of the health system and the marginalisation of people living with severe mental illness.^6^ A number of recent reforms in Australia, such as the establishment of mental health commissions, provide opportunities for the type of public oversight and analysis of their use that is occurring in the UK.

If the fundamental research challenge is to establish better the significance of CTOs, this is likely to be contested and therefore there is a genuine role for public debate. By its nature, such a discourse will have multiple perspectives. It necessitates a more pluralistic approach to constructing the necessary knowledge of CTOs to enable communities to make sound decisions about their use.
